# Nuclear Receptor Nur77 Controls Cardiac Fibrosis through Distinct Actions on Fibroblasts and Cardiomyocytes

**DOI:** 10.3390/ijms22041600

**Published:** 2021-02-05

**Authors:** Lejla Medzikovic, Hylja Heese, Pieter B. van Loenen, Cindy P. A. A. van Roomen, Ingeborg B. Hooijkaas, Vincent M. Christoffels, Esther E. Creemers, Carlie J. M. de Vries, Vivian de Waard

**Affiliations:** 1 Department of Medical Biochemistry, Amsterdam University Medical Centers (Amsterdam UMC), Location Academic Medical Center (AMC), Amsterdam Cardiovascular Sciences, University of Amsterdam, 1105 AZ Amsterdam, The Netherlands; lmedzikovic@ucla.edu (L.M.); hyljaheese@hotmail.com (H.H.); PietervanLoenen@outlook.com (P.B.v.L.); c.vanroomen@amsterdamumc.nl (C.P.A.A.v.R.); c.j.devries@amsterdamumc.nl (C.J.M.d.V.); 2 Department of Anesthesiology and Perioperative Medicine, Division of Molecular Medicine, David Geffen School of Medicine at University of California Los Angeles, Los Angeles, CA 90095, USA; 3 Department of Medical Biology, Amsterdam UMC, Location AMC, Amsterdam Cardiovascular Sciences, University of Amsterdam, 1105 AZ Amsterdam, The Netherlands; ingeborg.hooijkaas@gmail.com (I.B.H.); v.m.christoffels@amsterdamumc.nl (V.M.C.); 4 Department of Experimental Cardiology, Amsterdam UMC, Location AMC, Amsterdam Cardiovascular Sciences, University of Amsterdam, 1105 AZ Amsterdam, The Netherlands; e.e.creemers@amsterdamumc.nl

**Keywords:** cardiac, fibroblast, myofibroblast, fibrosis, nuclear receptor, cardiomyocyte, transforming growth factor β

## Abstract

Fibrosis is a hallmark of adverse cardiac remodeling, which promotes heart failure, but it is also an essential repair mechanism to prevent cardiac rupture, signifying the importance of appropriate regulation of this process. In the remodeling heart, cardiac fibroblasts (CFs) differentiate into myofibroblasts (MyoFB), which are the key mediators of the fibrotic response. Additionally, cardiomyocytes are involved by providing pro-fibrotic cues. Nuclear receptor Nur77 is known to reduce cardiac hypertrophy and associated fibrosis; however, the exact function of Nur77 in the fibrotic response is yet unknown. Here, we show that Nur77-deficient mice exhibit severe myocardial wall thinning, rupture and reduced collagen fiber density after myocardial infarction and chronic isoproterenol (ISO) infusion. Upon Nur77 knockdown in cultured rat CFs, expression of MyoFB markers and extracellular matrix proteins is reduced after stimulation with ISO or transforming growth factor–β (TGF-β). Accordingly, Nur77-depleted CFs produce less collagen and exhibit diminished proliferation and wound closure capacity. Interestingly, Nur77 knockdown in neonatal rat cardiomyocytes results in increased paracrine induction of MyoFB differentiation, which was blocked by TGF-β receptor antagonism. Taken together, Nur77-mediated regulation involves CF-intrinsic promotion of CF-to-MyoFB transition and inhibition of cardiomyocyte-driven paracrine TGF-β-mediated MyoFB differentiation. As such, Nur77 provides distinct, cell-specific regulation of cardiac fibrosis.

## 1. Introduction

Heart failure (HF) may be caused by acute cardiac injury, such as myocardial infarction (MI) or by chronic stressors, including adrenergic overstimulation [1,2]. HF is preceded by adverse cardiac remodeling, which is characterized by excessive deposition of extracellular matrix (ECM) proteins [3]. Given the limited regenerative capacity of the heart, fibrosis is an important repair process to preserve ventricle geometry and manage altered mechanical forces to prevent cardiac rupture [4]. However, excessive fibrosis reduces myocardial compliance and thus promotes HF [5]. Therefore, a balanced fibrotic response is crucial to maintain cardiac function after injury.

Fibroblasts are key players in maintaining ECM homeostasis. In the healthy heart, fibroblasts are present throughout the myocardium and become activated upon cardiac stress [6,7]. Activated cardiac fibroblasts (CF) proliferate and differentiate into so-called myofibroblasts (MyoFB). MyoFBs exhibit an excessive synthesis of ECM proteins to promote wound healing [3]. A variety of stimuli, such as chronic sympathetic hyperactivity, may induce MyoFB differentiation [7,8,9]. In addition, paracrine signals secreted from stressed cardiomyocytes play an important role in CF activation [10,11]. Transforming growth factor-β (TGF-β) is proposed as a central mediator of MyoFB differentiation in cardiac fibrosis [12].

Nur77 is a nuclear receptor involved in stress responses in various cell types and disease models [13,14,15]. Nur77 has been reported to attenuate skin and pulmonary fibrosis [16], vocal cord fibrosis [17] and endometrial fibrosis [18] by acting as an inhibitor of TGF-β signaling in fibroblasts. However, these observations have been challenged by reports showing that Nur77 promotes TGF-β signaling in embryonic fibroblasts [19] and breast cancer cells [19,20]. Nur77 has been emerging as an important regulator of cardiac hypertrophy and fibrosis by affecting the function of distinct cell types present in the heart. Indeed, Nur77 regulates cardiomyocyte Ca^2+^ homeostasis [21,22], apoptosis [23], infiltration of inflammatory monocytes into the injured myocardium [24] and cardiac sympathetic stimulation [25]. At present, the functional role of Nur77 in CF, MyoFB and the fibrotic response remains unknown. Here, we report that Nur77 promotes the differentiation of CFs into MyoFB, whereas in cardiomyocytes, Nur77 inhibits TGF-β expression to limit paracrine MyoFB differentiation. Together these data reveal the intricate regulatory function of Nur77 in cardiac fibrosis.

## 2. Results

### 2.1. Cardiac Ruptures and Reduced Cardiac Scar Density Are Observed in Nur77-KO Mice

**Figure 1:**
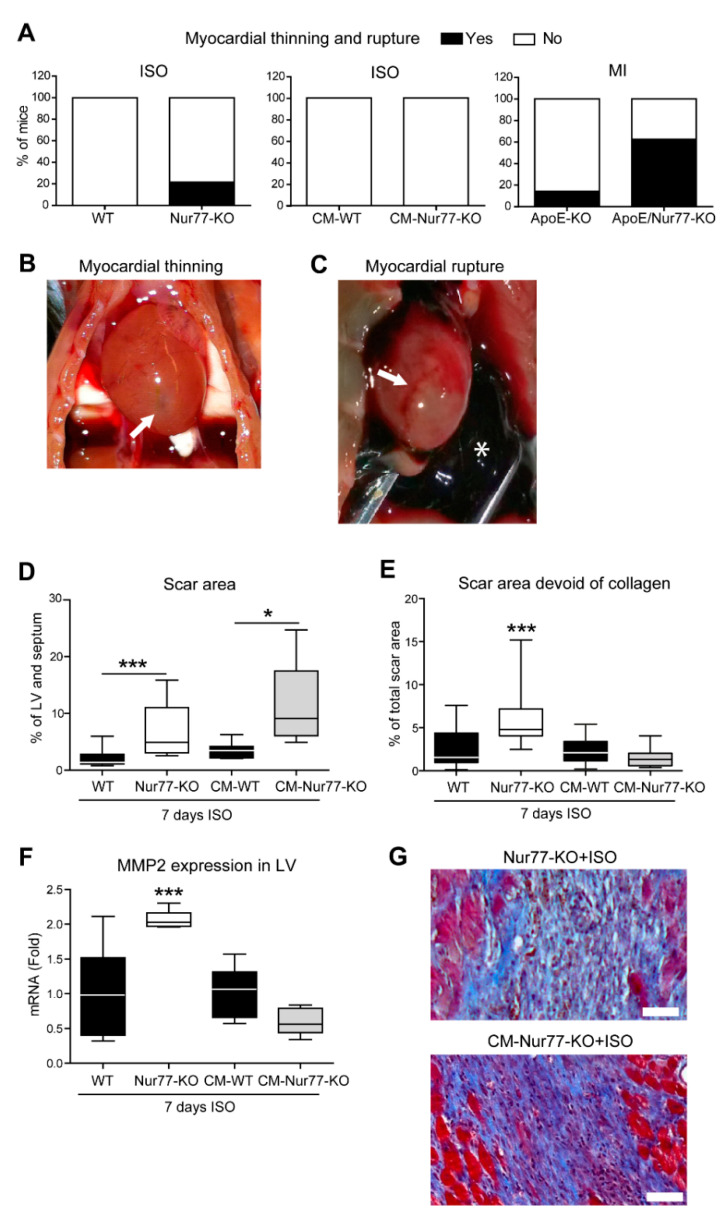
Cardiac ventricular wall thinning, rupture and reduced cardiac scar density in Nur77-KO mice. (**A**) Incidence of myocardial wall thinning and rupture in full-body Nur77-KO, full-body ApoE/Nur77-KO mice, and cardiomyocyte-specific Nur77-KO (CM-KO) mice after 2 weeks of permanent LAD ligation or 7 days of chronic isoproterenol (ISO, 60 mg/kg/day) infusion. (**B**) A typical example of severe myocardial wall thinning (arrow). (**C**) A typical example of cardiac rupture (arrow) with a blood clot in the chest cavity (asterisk). (**D**) Area of the left ventricle (LV) and septum affected by fibrosis upon 7 days of isoproterenol (ISO, 60 mg/kg/day) infusion quantified on Masson trichrome-stained tissue sections. (**E**) Histologic quantification of empty space between collagen fibrils in cardiac fibrotic patches on Masson trichrome-stained heart sections. (**F**) Expression levels of matrix metalloproteinase 2 (MMP2) in LV as assessed by qPCR. *n* = 8–20 mice per group. (**G**) Typical examples of cardiac fibrotic patches stained with Masson trichrome. Blue is collagen. Data presented as boxplots with whiskers for minimum/maximum values; * *p* < 0.05, *** *p* < 0.001.

The fibrotic response of the heart after insults, such as MI or chronic stress, is crucial to maintain the integrity of the heart and prevent acute rupture [26,27,28]. At the same time, excessive fibrosis interferes with proper diastolic and systolic function and electrical conductance, illustrating the importance of optimal regulation of the fibrotic response. We observed that chronic isoproterenol (ISO) stimulation caused cardiac rupture and severe myocardial thinning in Nur77-deficient (Nur77-KO) mice (5 ruptures/thinning in 23 mice; 21.74%), whereas this was not observed in wild-type (WT) mice (0/20; Figure 1A). Typical examples of severe myocardial thinning and cardiac rupture (arrows) with a blood clot in the chest cavity (asterisk) are shown in Figure 1B,C, respectively. The cardiac wall thinning was observed upon perfusion of the heart, when the needle in the apex was visible through the thin cardiac wall. These observations suggest a defect in the fibrotic response in Nur77-KO mice. We next assessed the effect of Nur77-deficiency on cardiac wall thinning and rupture after MI by left anterior descending coronary artery (LAD) ligation in mice on an apolipoprotein E (ApoE)-deficient background. Likewise, in response to MI, ApoE/Nur77-KO mice exhibited macroscopically visible myocardial thinning or cardiac rupture at a higher incidence (5/8; 62.5%) than ApoE-KO mice (1/7; 14.29%) (Figure 1A). Interestingly, in mice deficient for Nur77, specifically in cardiomyocytes (CM-KO mice), myocardial thinning/rupture did not occur upon chronic ISO infusion (Figure 1A), suggesting a major role for cardiac fibroblasts (CFs) in the fibrotic response. It is already known that Nur77 deficiency in monocytes and macrophages plays a role in the outcome of fibrotic scar size and density after LAD ligation [24]. Moreover, hypercholesterolemic mice have a higher incidence of cardiac rupture than normocholesterolemic mice [29]. Therefore, we continued to assess cardiac rupture in Nur77-KO mice upon chronic ISO stimulation, limiting the influence of inflammatory cells and hypercholesterolemic background.

Remarkably, Nur77-KO and CM-KO mice both exhibited larger fibrotic areas compared to their WT controls after ISO stimulation to a similar extent between the two genotypes (Figure 1D) [21,25]. Since the scar size is similar, but the total body Nur77-KO mice suffer from cardiac events, a difference in composition of the cardiac fibrotic patches between full-body Nur77-KO and CM-specific Nur77-KO mice may explain myocardial thinning and rupture in the Nur77-KO. To substantiate this hypothesis, we measured the density of the collagen matrix in cardiac fibrotic areas. We found that fibrotic areas in WT and CM-KO hearts exhibited similar collagen densities, while Nur77-KO mice had significantly more empty space between collagen fibrils, indicating loss of fiber quality or alignment (Figure 1E). This difference was further highlighted by elevated expression levels of matrix metalloproteinase 2 (MMP2; Figure 1F) only in Nur77-KO mouse LV. Typical examples of different cardiac fibrotic patch morphologies are shown in Figure 1G.

### 2.2. Nur77 Knockdown in CFs Represses MyoFB Marker Expression

**Figure 2:**
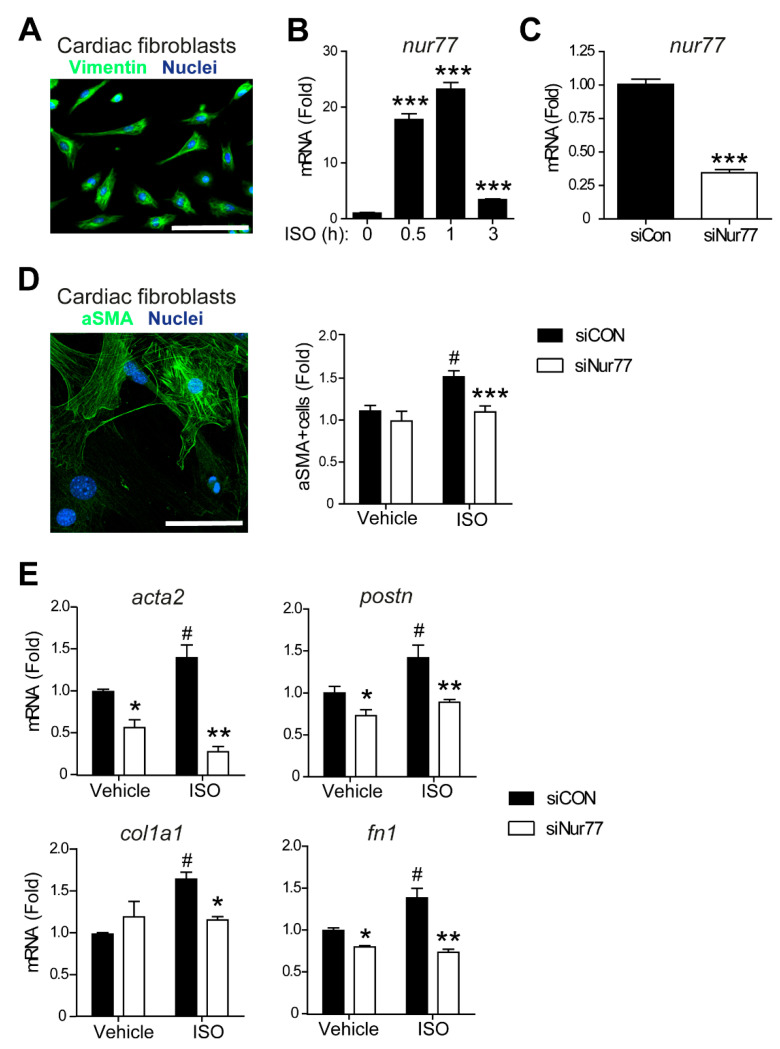
Nur77 knockdown in CFs promotes a MyoFB phenotype. (**A**) Primary neonatal rat CF in culture were identified by expression of fibroblast marker vimentin. Scale bar represents 100 μm. (**B**) Induction of Nur77 mRNA expression in CFs after ISO (10 μM) treatment. (**C**) Decreased Nur77 mRNA expression after siRNA-mediated knockdown in CFs (siNur77) compared to CFs treated with control siRNA (siCon), as measured by qPCR. (**D**) Number of CF expressing MyoFB marker α-smooth muscle actin (aSMA) as assessed by immunofluorescence. Scale bar represents 20 μm. (**E**) MyoFB and ECM-related gene expression measured by qPCR. *acta2*: α-smooth muscle actin, *col1a1*: collagen type 1, *fn1*: fibronectin, *postn*: periostin. (**D**,**E**) ISO stimulation (10 μM) was for 24 h. *n* = 3–4 independent experiments. Data presented as mean + SEM; (**B**): *** *p* < 0.001 vs. t = 0; (**C**–**E**): # *p* < 0.05 vs. siCon vehicle; * *p* < 0.05, ** *p* < 0.01, *** *p* < 0.001 vs. siCon same stimulus.

Both cardiomyocytes and CFs contribute to the cardiac fibrotic response [30]. Since MyoFB are the main mediators of fibrosis in the remodeling heart [6], we assessed the role of Nur77 in CF-to-MyoFB transition in response to ISO. Cultured neonatal rat CF were identified by expression of vimentin (Figure 2A). First, we stimulated neonatal rat CFs with ISO and observed that Nur77 mRNA is rapidly upregulated (Figure 2B), indicating functional involvement of Nur77 in CFs. To assess the function of Nur77 in CF-to-MyoFB transition, we performed siRNA-mediated knockdown of Nur77 (siNur77-CFs, Figure 2C). ISO treatment induced MyoFB transition as illustrated by an enhanced number of CF expressing MyoFB marker α-smooth muscle actin (αSMA) (Figure 2D) [31]. Interestingly, ISO did not significantly increase the number of αSMA-expressing CF upon Nur77 knockdown (Figure 2D), indicating inhibition of MyoFB transition by Nur77 knockdown. We next assessed the ISO-induced MyoFB phenotype at the gene expression level (Figure 2E). Genes associated with the MyoFB phenotype, αSMA (*acta2*) and periostin (*postn*) were expressed to a significantly higher extent in siCon CFs upon ISO stimulation, whereas siNur77 CFs did not show this increased expression. Additionally, expression of genes encoding ECM proteins such as type 1α collagen (*col1a1*) and fibronectin (*fn1*) was lower in ISO-stimulated siNur77-CFs compared to siCon-CFs (Figure 2E). Interestingly, *acta2*, *postn* and *fn1* were already differentially expressed between siCon and siNur77-CFs under control conditions. Together, these data support that Nur77 enhances ISO-induced CF-to-MyoFB transition.

### 2.3. Nur77 Knockdown in CFs Represses MyoFB Functional Characteristics

**Figure 3:**
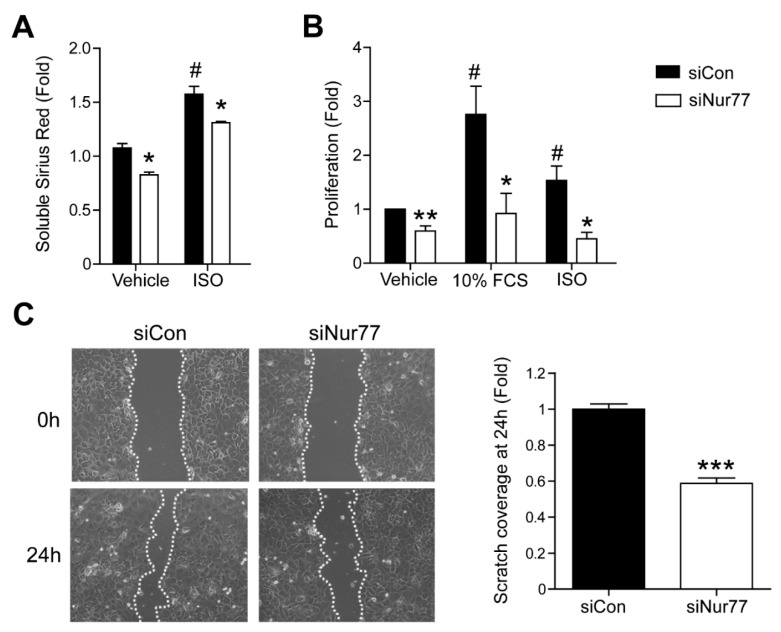
Nur77 knockdown in cardiac fibroblasts (CFs) represses MyoFB functional characteristics in CF. (**A**) CF collagen content as measured by soluble Sirius red assay. (**B**) CF proliferation as measured by bromodeoxyuridine (BrdU) incorporation. (**C**) CF wound closure capacity in scratch wound assay; quantification in the right panel. (**A**) ISO (10 μM) stimulation was for 72 h. (**B**) ISO (10 μM) stimulation was for 24 h. *n* = 3–4 independent experiments per group. Data presented as mean + SEM; # *p* < 0.05 vs. siCon vehicle; * *p* < 0.05, ** *p* < 0.01, *** *p* < 0.001 vs. siCon same stimulus.

We next studied the effect of Nur77 on MyoFB function. MyoFBs synthesize and deposit elevated levels of collagen and proliferate more than quiescent CFs, leading to exaggerated and aberrant wound healing [3]. In accordance with an attenuated MyoFB marker expression profile, siNur77 CFs produce less collagen (Figure 3A). Lower collagen content in siNur77 CFs was observed in non-stimulated conditions, as well as after stimulation with ISO. Furthermore, siNur77 CFs proliferate significantly less than siCon CFs at baseline and proliferation was no longer induced by fetal calf serum or ISO after Nur77 knockdown (Figure 3B). Finally, siNur77 CFs possess lower wound closure capacity in the scratch-wound assay compared to siCon CFs (Figure 3C). These results indicate that, in addition to promoting MyoFB differentiation on marker expression level, Nur77 also enhances MyoFB collagen production, proliferation and wound closure capacity on a functional level.

### 2.4. Paracrine Factors from Nur77-Silenced Cardiomyocytes Promote MyoFB Differentiation

**Figure 4:**
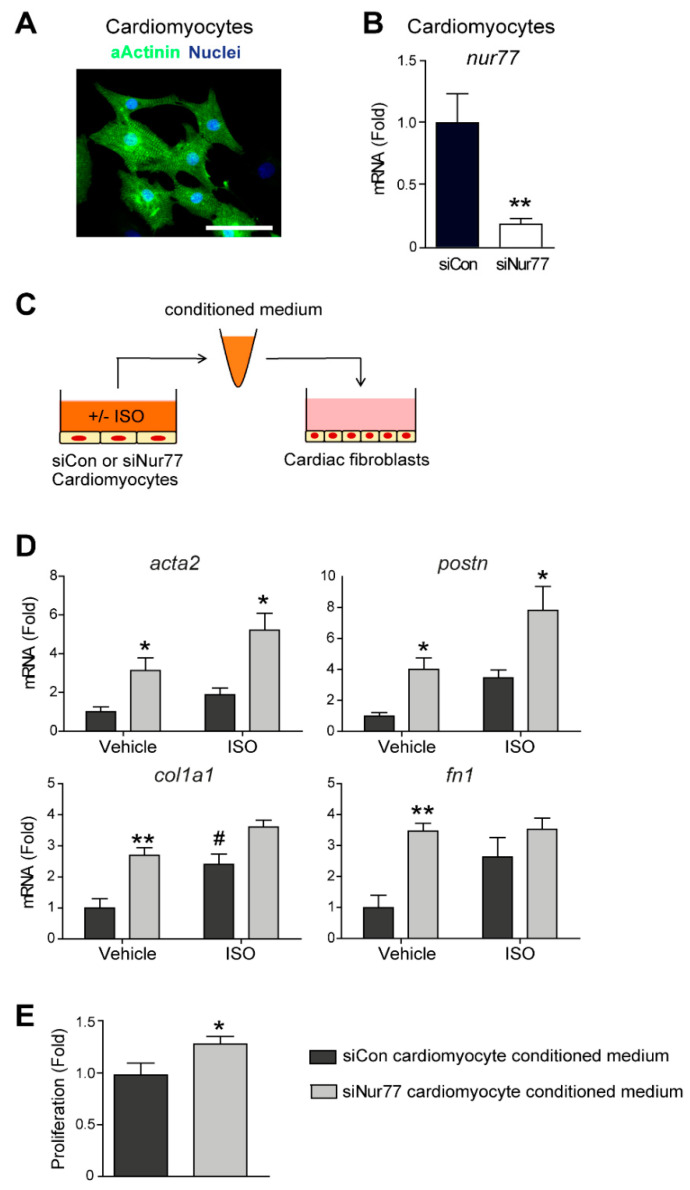
Paracrine factors from Nur77-silenced cardiomyocytes promote a MyoFB phenotype in CF. (**A**) Primary neonatal rat ventricular cardiomyocytes in culture were identified by expression of α-actinin. Scale bar represents 100 μm). (**B**) Nur77 mRNA expression after siRNA-mediated knockdown of Nur77 in neonatal rat ventricular cardiomyocytes, as measured by qPCR. (**C**) Schematic presentation of the experimental setup. (**D**) mRNA expression of MyoFB markers in CF after incubation with siCon or siNur77 cardiomyocyte conditioned medium, as assessed by qPCR. (**E**) Proliferation of CF after incubation with siCon or siNur77 cardiomyocyte conditioned medium, as measured by BrdU incorporation. (**D**,**E**) Conditioned medium stimulation (1:2 ratio with fresh medium) was for 24 h. *n* = 3–4 independent experiments. Data presented as mean + SEM; # *p* < 0.05 vs. siCon vehicle; * *p* < 0.05, ** *p* < 0.01 vs. siCon same stimulus.

During adverse cardiac remodeling, CFs become activated directly by pathological stimuli, but CFs are also affected by pro-fibrotic factors that are secreted by stressed cardiomyocytes [30]. Cardiomyocytes are known to secrete such factors upon ISO stimulation [11]. We have previously shown that Nur77 knockdown in cardiomyocytes leads to enhanced ISO-induced hypertrophy [21]. Therefore, we next assessed the role of Nur77 in cardiomyocyte-mediated CF activation. We identified neonatal rat ventricular cardiomyocytes (NRCM) in culture by the expression of cardiomyocyte marker α-actinin (Figure 4A). Next, we knocked down Nur77 in NRCM (Figure 4B), stimulated the NRCMs with ISO and subsequently transferred the conditioned medium to CFs (Figure 4C). Strikingly, we found that the expression of MyoFB-associated and ECM genes *acta2*, *postn*, *col1a1* and *fn1* was significantly higher in CFs stimulated with siNur77-NRCM conditioned medium (Figure 4D). This difference seemed to originate from Nur77-silencing in NRCMs since ISO stimulation did not cause an additive effect on MyoFB gene expression, except for *col1a1* expression. Additionally, CFs proliferated significantly more when stimulated with siNur77 NRCM conditioned medium compared to medium from siCon NRCMs (Figure 4E). Together, these results show that Nur77 reduces the expression of pro-fibrotic paracrine factors in NRCMs and reveal that Nur77 regulates an intricate balance of CF-to-MyoFB differentiation, with seemingly opposing roles by cardiomyocytes and CFs in fibrosis.

### 2.5. Nur77-Silenced Cardiomyocytes Promote a MyoFB Phenotype via TGF-β

**Figure 5:**
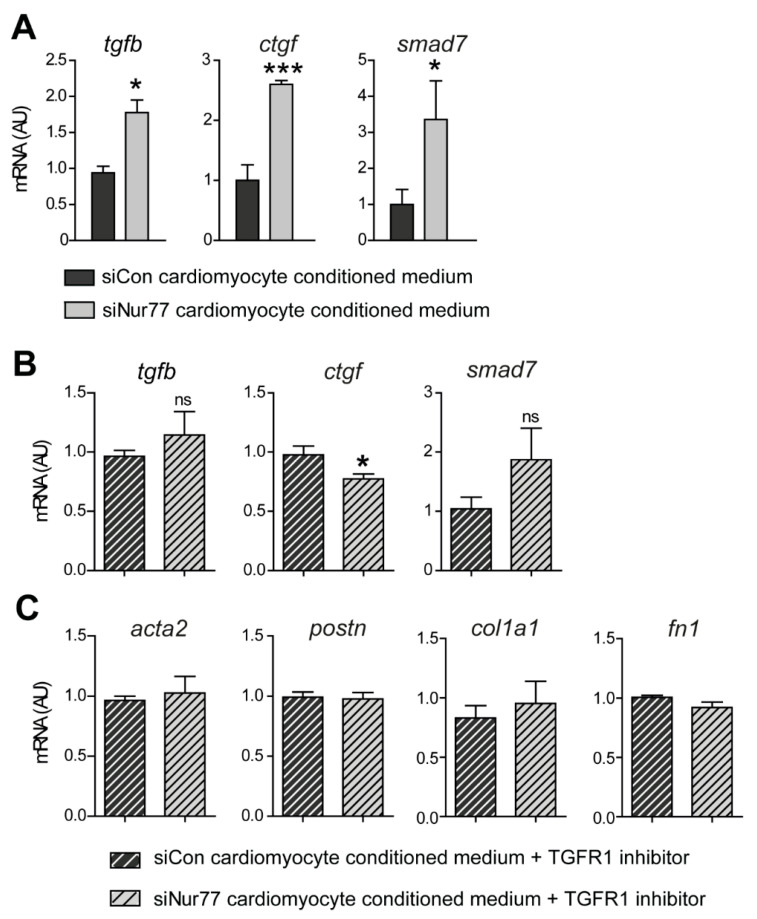
Nur77-silenced cardiomyocytes promote a MyoFB phenotype in CF via TGF-β. (**A**) Expression of TGF-β and target genes in CF stimulated with conditioned medium from siCon or siNur77 neonatal rat cardiomyocytes, as assessed by qPCR. (**B**) Expression of TGF-β target genes and MyoFB genes (**C**) in CFs stimulated with conditioned medium from siCon or siNur77 neonatal rat cardiomyocytes after 15 min pre-incubation with 10 μM TGF-β receptor I (TGFR1) inhibitor SB431542. Conditioned medium stimulation (1:2 ratio with fresh medium) was for 24 h. *tgfb*: transforming growth factor—β, *ctgf*: connective tissue growth factor. Data presented as mean + SEM; * *p* < 0.05, *** *p* < 0.001 vs. siCon same stimulus, ns—not significant.

A major paracrine factor regulating communication between cardiomyocytes and CFs in the setting of cardiac fibrosis is TGF-β [30]. Nur77 is known to regulate TGF-β signaling in various cell types [16,18,19,20]. Therefore, we assessed the expression of *tgfb* and classical TGF-β target genes *ctgf* and *smad7* in CFs upon stimulation with siNur77 NRCM conditioned medium. We found significantly upregulated expression of *tgfb*, *ctgf* and *smad7* in CFs treated with siNur77 NRCM conditioned medium compared to CFs treated with siCon NRCM medium (Figure 5A). To confirm that TGF-β was the main pro-fibrotic factor in the siNur77 NRCM conditioned medium, we next pre-incubated CFs with TGF-β receptor I inhibitor to block TGF-β signaling before stimulation with NRCM conditioned medium. Indeed, the expression of TGF-β target genes (Figure 5B), MyoFB genes and ECM genes (Figure 5C) did no longer differ between CFs stimulated with siCon NRCM or siNur77 NRCM conditioned medium, hence rescuing the pro-fibrotic phenotype of the siNur77-NRCM conditioned medium.

### 2.6. Nur77 Silencing in CF Inhibits TGF-β-Induced Signaling and MyoFB Transition

**Figure 6:**
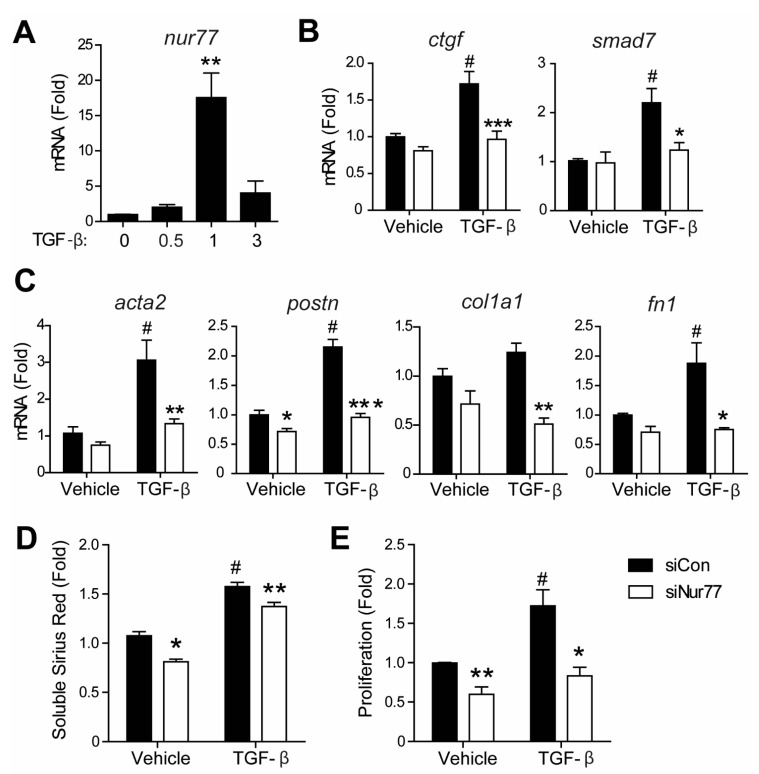
Nur77 knockdown in CFs inhibits TGF-β-induced signaling and myofibroblasts (MyoFB) differentiation. (**A**) Induction of Nur77 mRNA in CFs after TGF-β (10 ng/mL) stimulation. (**B**) Expression of TGF-β pathway downstream markers in siCon-CFs and siNur77-CFs stimulated with TGF-β, as measured by qPCR. (**C**) Expression of MyoFB markers in siCon-CFs and siNur77-CFs stimulated with TGF-β, as measured by qPCR. (**D**) CF collagen production as measured by soluble Sirius red assay. (**E**) CF proliferation as measured by BrdU incorporation. (**B**,**C**,**E**) TGF-β stimulation (10 ng/mL) was for 24 h. (**D**) TGF-β stimulation (10 ng/mL) was for 72 h. *n* = 3–4 independent experiments per group. Data presented as mean + SEM; (**A**): ** *p* < 0.01 vs. t = 0; (**B**–**D**): # *p* < 0.05 vs. siCon vehicle; * *p* < 0.05, ** *p* < 0.01, *** *p* < 0.001 vs. siCon TGF-β.

After elucidating that Nur77 in cardiomyocytes represses MyoFB transition by reducing TGF-β secretion, we next assessed the function of Nur77 in TGF-β-mediated signaling in CFs. Nur77 is induced rapidly and robustly in CFs by TGF-β (Figure 6A). The expression of TGF-β target genes (Figure 6B), MyoFB genes, and ECM genes (Figure 6C) were significantly lower in siNur77 CFs compared to siCon CFs. In line with reduced MyoFB transition, siNur77 CFs produced less collagen, and proliferation was lower in response to TGF-β compared to siCon CFs (Figure 6C,D).

**Figure 7:**
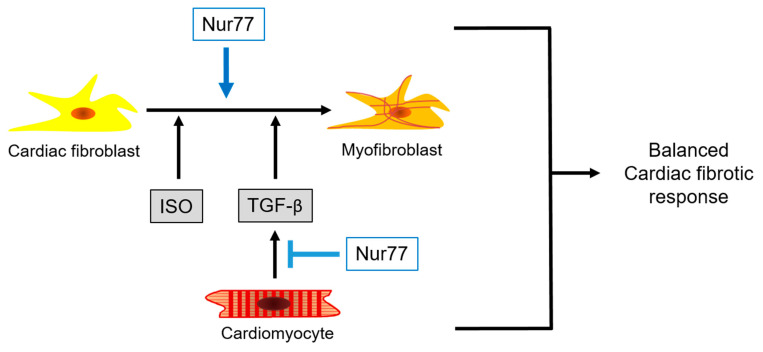
Proposed working model. Nur77 in cardiac fibroblasts promotes ISO and TGF-β-induced fibroblast-to-myofibroblast transition, while Nur77 in cardiomyocytes represses the ability of these cells to induce paracrine TGF-β-mediated fibroblast-to-myofibroblast transition, thereby balancing the cardiac fibrotic response. ISO: isoproterenol, TGF-β: transforming growth factor-β.

Together our in vitro data indicate that Nur77 in CFs promotes ISO and TGF-β-induced CF-to-MyoFB transition, while Nur77 in cardiomyocytes represses the ability of these cells to induce paracrine TGF-β-mediated CF-to-MyoFB transition (Figure 7), revealing a mechanism to balance the cardiac fibrotic response. Our data support the observed differential cardiac fibrosis phenotype in Nur77-KO and CM-KO mice, while in the Nur77-KO mice, both cardiomyocytes and fibroblasts lack Nur77, resulting in aberrant scar formation.

## 3. Discussion

Fibrosis is a hallmark of adverse cardiac remodeling, which promotes HF by reducing myocardial compliance [5]. However, fibrotic scar formation is essential to allow cardiac repair upon injury and to accommodate altered mechanical forces to prevent rupture [4]. Therefore, cardiac fibrosis requires intricate regulation at specific moments after a cardiac insult, allowing tissue healing and resolving the scars. Unraveling the underlying mechanism is challenging because several different cell types are involved, among which cardiac (myo)fibroblasts and cardiomyocytes, but also different subsets of infiltrating monocytes and macrophages play an important role in myocardial repair after MI [32,33].

We aimed to understand the role of Nur77 in cardiac fibrosis. Nur77 is a nuclear receptor expressed in all cardiac cell types in response to acute stressors. As a measure for an inadequate fibrotic response, we determined cardiac rupture and macroscopically visible wall thinning in dedicated mouse models. More ApoE/Nur77-KO mice exhibited myocardial thinning and rupture after MI than ApoE-deficient mice. It has been shown that Nur77 deficiency in monocytes and macrophages promotes a proinflammatory phenotype, leading to impaired myocardial repair and larger scar size with reduced collagen density after MI [24,33]. Furthermore, Nur77 was shown to repress endothelial-to mesenchymal transition, leading to enhanced MI-induced fibrotic scar size in Nur77-KO mice [34]. Additionally, epicardial cells are thought to be involved in myocardial repair responses after MI by giving rise to cardiac myofibroblasts through epithelial-mesenchymal transition [7,35]. While the role of Nur77 in epicardial cells was not studied here, it may also be of interest in relation to the fibrotic response and rupture after MI in Nur77-KO mice, since we have observed high Nur77 expression in epicardial cells upon MI in mice (data not shown). We furthermore cannot exclude the influence of proinflammatory macrophages and endothelial cells on myocardial thinning and rupture in our MI experiments with ApoE/Nur77-KO mice [24,34,36]. However, in the one-week model of ISO-induced cardiac hypertrophy, where monocyte infiltration, macrophage accumulation, and the expression of proinflammatory genes are not prominent, Nur77-KO mice also exhibit severe myocardial thinning, rupture and reduced scar density. The mere fact that cardiomyocyte-specific Nur77 deficient mice, the CM-KO, develop increased cardiac fibrosis to the same extent as whole-body Nur77-KO mice, but that only the Nur77-KO hearts show an aberrant collagen fiber structure, made us the reason that Nur77 is involved in regulating the interplay between (myo)fibroblasts and cardiomyocytes in fibrosis. Based on our data, we conclude that Nur77 modulates MyoFB differentiation in the heart by diverse mechanisms. In CFs, Nur77 enhances differentiation into MyoFBs upon stimulation with either ISO or TGF-β. In cardiomyocytes, however, Nur77 represses the ability of these cells to induce TGF-β–mediated paracrine MyoFB differentiation. This imbalance in cell-specific TGF-β expression and signaling may underlie the impaired cardiac fibrotic response in full-body Nur77-KO mice.

The canonical TGF-β signaling pathway acts via phosphorylation of SMAD2/3 transcription factors that induce subsequent expression of MyoFB genes [37]. Nur77 has been reported to potentiate canonical TGF-β signaling by facilitating the ubiquitination and degradation of SMAD7, a potent inhibitor of TGF-β signaling. In Nur77-KO mouse embryonic fibroblasts, this leads to decreased TGF-β–induced phospho-SMAD2 levels and expression of downstream MyoFB genes [19], which is in line with our results in siNur77 CFs. In cancer cells, Nur77 silencing inhibits the phospho-SMAD3 expression and transcriptional activity in response to TGF-β. Concomitantly, migration of these cells is lower upon Nur77 silencing [19]. Altered TGF-β signaling may mediate the opposing actions of Nur77 in CFs and cardiomyocytes since recently; it has been shown that SMAD3 signaling in cardiomyocytes and cardiac fibroblasts has different effects on cardiac remodeling post-MI. In this model, CF SMAD3 signaling promotes scar organization by integrin synthesis, while cardiomyocyte SMAD3 signaling induces MMP activation [38]. This is especially interesting as we have previously shown that Nur77 regulates the expression of various MMPs [39,40], and we show that MMP2 expression is upregulated in LV of ISO-treated Nur77-KO mice, but not CM-KO or WT. Whether this TGF-β/SMAD3/MMP pathway underlies the reduced scar density and enhanced ruptures in Nur77-KO mice, and whether it predominantly originates from CF/MyoFB or cardiomyocytes remains to be elucidated. Future co-culture and paracrine signaling experiments employing Nur77-deficient CF and cardiomyocytes, as well as the generation of fibroblast-specific Nur77-KO mouse models, will further elucidate the role of Nur77 in the interplay between these cardiac cells in the cardiac fibrotic response.

To the best of our knowledge, this is the first study to report on the functional role of Nur77 in cardiac CF to MyoFB transition and in the fibrotic cues synthesized by cardiomyocytes. Together, our results support the hypothesis that Nur77 acts as a modifier gene in adverse cardiac remodeling by regulating the fibrotic response in both cardiomyocytes and CFs.

## 4. Methods

### 4.1. Animal Experiments

All animal care procedures and experiments were approved by the Institutional Animal Ethics Committee of the University of Amsterdam (Approval numbers 17-1804-1-1; 102967-1 01-01-2014; DBC54AG 12-12-2016; DBC54AH 28-02-2017), in accordance with institutional and European directive 2010/63/EU guidelines.

### 4.2. LAD Ligation

C57Bl6/J ApoE-KO mice (stock #002052) and Nur77-KO (stock #006187) mice were purchased from The Jackson Laboratory and crossed to obtain ApoE/Nur77-KO mice. These Nur77-KO have been used globally for decades, yet it is good to realize that these mice still produce an amino-terminal domain of Nur77 [41]. Mice were switched to a Western-type diet (Arie Blok, Woerden, The Netherlands) two weeks prior to experiments. Male, 10–14 week–old mice were subjected to permanent ligation of the left anterior descending (LAD) coronary artery, under isoflurane anesthesia (4% isoflurane for induction, 2% isoflurane and O_2_ for maintenance of anesthesia; Baxter) with Temgesic as an analgesic. Mice were monitored twice daily for humane endpoints or sudden death. After 14 days, the mice were euthanized through a lethal dose of ketamine (166 mg/kg)/xylazine (23.8 mg/kg) injected intraperitoneally, and hearts were excised.

### 4.3. In Vivo Isoproterenol-Induced Fibrosis

WT, Nur77-KO, cardiomyocyte-specific Nur77-deficient mice (CM-KO) and their control littermates (CM-WT) were employed in previous studies [21,25]. Briefly, osmotic minipumps (Alzet, Cupertino, CA, USA) were implanted subcutaneously under isoflurane anesthesia (4% isoflurane for induction, 2.5% isoflurane and O_2_ for maintenance of anesthesia; Baxter, IL, United States) with subcutaneously administered carprofen (4 mg/kg) as a local analgesic. Osmotic minipumps contained 60 mg/kg/day isoproterenol (Sigma, St. Louis, MO, USA) or saline as control. After 7 days, the mice were euthanized through a lethal dose of intraperitoneal-injected ketamine (238 mg/kg)/xylazine (102 mg/kg), after which hearts were excised.

### 4.4. Autopsy

Autopsies were performed on animals that were found dead, reached humane endpoints or were sacrificed at the end of the study. Myocardial thinning was determined by macroscopic inspection of the heart. Death by cardiac rupture was characterized by a large blood clot around the heart and/or in the chest cavity.

### 4.5. Collagen Density Measurements

Hearts were fixed in formalin, embedded in paraffin, and heart tissue sections were stained with Masson trichrome, as previously reported [21]. Using Leica QWin V3 software (Leica Microsystems, Cambridge, UK), the ratio of the fibrotic area compared to the total septum and left ventricle area was quantified to define fibrotic scar area. To identify the scar area devoid of collagen, the ratio of empty space (white pixels) and collagen content (blue pixels) compared to the total area of the fibrotic patch (white and blue pixels together) was determined.

### 4.6. Neonatal Rat Cardiomyocyte (NRCM) and Cardiac Fibroblast (CF) Isolation, Culture, and Stimulation

NRCMs were isolated as described previously [21]. Briefly, hearts from 1–3 day old Wistar rats were excised, and atria were removed. The heart was cut into pieces and incubated overnight, rotating at 4 °C in HBSS (Invitrogen, Carlsbad, CA, USA) containing 1 mg/mL trypsin (Affymetrix, Santa Clara, CA, USA). The next day, cells were dissociated using 1 mg/mL collagenase (Worthington, Lakewood, NJ, USA) in HBSS. Cells were collected and resuspended in M199 culture medium, supplemented with 1% HEPES, 1% NEAA, 2 mg/L vitamin B12, 3.5 g/L glucose, and antibiotics (all from Invitrogen), and pre-plated to separate myocytes from fibroblasts. After 2 h, non-adherent cells, the CMs, were collected and plated on fibronectin. Adherent cells were CFs.

NRCM was maintained in serum-free (Dulbecco’s modified Eagle’s medium (DMEM) containing 1% penicillin/streptavidin (Invitrogen) for 24 h before experiments. CF was grown in a fibroblast growth medium (DMEM with 10% fetal calf serum and 1% penicillin/streptavidin). CF from passages 2–3 was used for experiments. Before experiments, CF was starved in serum-free medium for 24 h. CFs were stimulated with saline, ISO (10 μM, Sigma) or TGF-β (10 ng/mL, R&D Systems, Minneapolis, MN, USA) as indicated.

### 4.7. Nur77 Knockdown

Nur77 was silenced by transfection of Nur77 siRNA (175 nM for NRCMs, 40 nM for CFs; SMARTpool, Dharmacon) using lipofectamine RNAiMAX (Invitrogen). A non-targeting control siRNA pool served as control. Transfections were carried out in serum-free Opti-MEM medium (Gibco, Gaithersburg, MD, USA) for 6 h. Cells were used for experiments 24 h after transfection.

### 4.8. RNA Isolation and qPCR

Total RNA was isolated from cells using Tri Reagent (Sigma), and cDNA was synthesized using iScript reverse transcription reagent (Bio-Rad, Veenendaal, The Netherlands) according to the manufacturer’s instructions. qPCR was performed on a Roche LightCycler with *rplp0* and *rpl13* serving as housekeeping genes. Primer sequences are listed in Table 1.

### 4.9. Immunofluorescence

Cells were fixed with 4% paraformaldehyde (Roth, Karlsruhe, Germany), blocked with 5% normal goat serum (Dako, Santa Clara, CA, USA) and permeabilized with 0.1% Triton X-100. Primary antibodies against vimentin (Abcam #ab27608), α-smooth muscle actin (Dako #M0851) or α-actinin (Sigma #A7811) were incubated overnight at 4 °C. The secondary antibody was Alexa488-conjugated (Invitrogen #A-11008 and #A-11001), and nuclei were stained with Hoechst (Invitrogen #H3570). Photomicrographs were taken with the EVOS cell imaging system, and positive cells were counted with ImageJ software.

### 4.10. Soluble Sirius Red Assay

Collagen content in CF was measured as described previously [40]. Briefly, CFs were stimulated with the indicated compounds for 72 h. Afterward, the culture medium was discarded, and the cells were fixed with 4% paraformaldehyde (Roth). To stain the collagen, cells were incubated with 0.1% Sirius red F3B dye (BDH Laboratory Supplies, Poole, UK) in 0.01 M HCl for 1 h. After extensive washing with 0.01 M HCl, the dye was dissolved in 0.01 M NaOH and absorbance was measured at OD550 in a microplate reader (EL808, Bio-Tek, Winooski, VT, USA). OD values were compared to a gelatin standard curve.

### 4.11. Proliferation Assay

Cells were stimulated with compounds as indicated, and simultaneously, BrdU was added. After 24 h, proliferation was assessed using the BrdU Cell Proliferation ELISA (Roche, Basel, Switzerland) according to the manufacturer’s instructions.

### 4.12. Scratch Wound Assay

After transfection, fibroblasts were grown to ~90% confluency, and a scratch was made using a p200 pipette tip where after the culture medium was refreshed. Pictures of the entire scratch were made using the EVOS FL Auto microscope (Thermo Fisher, Waltham, MA, USA) at t = 0 h and t = 24 h after the scratch was made. Using ImageJ, the surface area of the whole scratch wound at t = 0 h and t = 24 h was measured, and the ratio was used to calculate scratch wound coverage at 24 h.

### 4.13. Conditioned Medium Experiments

NRCMs were transfected and stimulated with saline or ISO (25 μM) for 48 h. Subsequently, a conditioned medium from NRCMs was collected and centrifuged to remove cell debris, whereafter it was snap-frozen in liquid nitrogen and stored at −80 °C until use. Conditioned medium was added to CF in a 1:2 ratio with fresh serum-free DMEM, and cells were incubated for 24 h. TGF-β receptor I inhibitor SB431542 (10 μM, Tocris, Bristol, UK) was added to the CFs 15 min before the addition of a conditioned medium.

### 4.14. Statistical Analysis

Analyses were performed using GraphPad Prism 5 software. Data distribution was tested with Kolmogorov–Smirnov normality test. Normally distributed data are presented as mean ± SEM and tested with Student’s *t*-test or one-way ANOVA with Holm–Bonferroni post hoc correction. Non-normally distributed data are presented as boxplots with whiskers for minimum/maximum values and tested with Kruskal–Wallis test with Dunn’s post hoc correction.

## Figures and Tables

**Table 1:** Primer sequences. *acta2*: α-smooth muscle actin, *col1a1*: collagen type 1, *ctgf*: connective tissue growth factor, *fn1*: fibronectin, *mmp2*: matrix metalloproteinase 2, *postn*: periostin, *rpl13*: ribosomal protein l13, *rplp0*: ribosomal protein p0, *tgfb*: transforming growth factor—β.

**Rat Primer**	**Forward**	**Reverse**
*acta2*	TTCAATGTCCCTGCCATGTA	GAAGGAATAGCCACGCTCAG
*col1a1*	TGCTGCCTTTTCTGTTCCTT	AAGGTGCTGGGTAGGGAAGT
*ctgf*	TAGCAAGAGCTGGGTGTGTG	TTCACTTGCCACAAGCTGTC
*fn1*	GAAAGGCAACCAGCAGAGTC	CTGGAGTCAAGCCAGACACA
*nur77*	TGTTGCTAGAGTCCGCCTTT	CAGTGATGAGGACCAGAGCA
*postn*	TCCTGAATACCCTCCAGTGC	AGGTCCGTGAAAGTGGTTTG
*rpl13*	AAAAAGGAGAAGGCCAGAGC	CCGCGCATTATTTCTTCTTC
*rplp0*	CTCAGTGCCTCACTCCATCA	CTTCCTTTGCTTCGACCTTG
*smad7*	TCCTGCTGTGCAAAGTGTTC	TCTGGACAGTCTGCAGTTGG
*tgfb*	ATACGCCTGAGTGGCTGTCT	TGGGACTGATCCCATTGATT
**Mouse primer**	**Forward**	**Reverse**
*mmp2*	GACCTTGACCAGAACACCATC	CATCCACGGTTTCAGGGTCC
*Rpl13*	GGGCAGGTTCTGGTATTGGAT	GGCTCGGAAGTGGTAGGGG
*Rplp0*	GGACCCGAGAAGACCTCCTT	GCACATCACTCAGAATTTCAATGG

## Data Availability

No data suitable for public databases were obtained.
